# Routine breast screening for women aged 65–69: results from evaluation of the demonstration sites

**DOI:** 10.1054/bjoc.2001.2047

**Published:** 2001-09-01

**Authors:** S M Moss, J Brown, L Garvican, D A Coleman, L E Johns, R G Blanks, G Rubin, J Oswald, A Page, A Evans, P Gamble, R Wilson, L Lee, J Liston, L Sturdy, G Sutton, G Wardman, J Patnick, R Winder

**Affiliations:** Cancer Screening Evaluation Unit, Section of Epidemiology, The Institute of Cancer Research, Block D, Cotswold Road, Sutton, SM2 5NG; MRC HSRC, Department of Social Medicine, University of Bristol, Canynge Hall, Whiteladies Road, Bristol, BS8 2PR; Honorary Research Fellow, Centre for Health Service Studies at Tunbridge Wells, University of Kent, Oak Lodge, David Salomon's Estate, Broomehill Road, Tonbridge Wells, Kent, TN3 0TG; Brighton and Hove Breast Screening Service; Helen Garrod Breast Screening Unit, City Hospital, Nottingham; Leeds and Wakefield Breast Screening Service; Wakefield Health Authority; Calderdale and Kirklees Health Authority; NHS Breast Screening Programme

**Keywords:** breast cancer, screening, older women

## Abstract

Routine programme data and specially designed surveys from 3 demonstration sites were analysed to determine the implications of extending the NHS Breast Screening Programme (NHSBSP), to include routine invitations for women up to 69 years. All women aged 65–69 and registered with GPs in these areas received routine invitations for breast screening along with those aged 50–64. Overall uptake was 71% in women aged 65–69 compared with 78% in younger women, but was ≥ 90% in both groups who had previously attended within 5 years. Recall rates were lower for older women, but with a higher positive predictive value for cancer. The percentages of invasive cancer in different prognostic categories were similar in the 2 age groups. Older women took no longer to screen than younger women. The costs per woman invited or per woman screened were also similar to those for women aged 50–64, whilst the cost per cancer detected was some 34% lower in older women. Breast screening is as cost effective for women aged 65–69 as for those aged 50–64, with a higher cancer detection rate balancing shorter life expectancy. The proposed extension to the national programme will have considerable workforce implications for the NHSBSP and require additional resources. © 2001 Cancer Research Campaign


*British Journal of Cancer* (2001) **85**(9), 1289–1294
© 2001 Cancer Research Campaign
doi: 10.1054/ bjoc.2001.2047, available online at http://www.idealibrary.com on IDEAL

### Routine breast screening for women aged 65–69: results from evaluation of the demonstration sites

**SM Moss[1], J Brown[2], L Garvican[3], DA Coleman[1], LE Johns[1], RG Blanks[1], G Rubin[4], J Oswald[4], A Page[4], A Evans[5], P Gamble[5], R Wilson[5], L Lee[5], J Liston[6], L Sturdy[6], G Sutton[7], G Wardman[8], J Patnick[9] and R Winder[9]**

[1]Cancer Screening Evaluation Unit, Section of Epidemiology, The Institute of Cancer Research, Block D, Cotswold Road, Sutton, Surrey SM2 5NG;[2]MRC HSRC, University of Bristol, Department of Social Medicine, Canynge Hall, Whiteladies Road, Bristol BS8 2PR; [3]Honorary Research Fellow, Centre for Health Service Studies at Tunbridge Wells, University of Kent, Oak Lodge, David Salomon's Estate, Broomehill Road, Tonbridge Wells, Kent TN3 0TG; East Sussex, Brighton and Hove Breast Screening Service; [5]Helen Garrod Breast Screening Unit, City Hospital, Nottingham; [6]Leeds and Wakefield Breast Screening Service; [7]Wakefield Health Authority; [8]Calderdale and Kirklees Health Authority; [9]NHS Breast Screening Programme

**Summary** Routine programme data and specially designed surveys from 3 demonstration sites were analysed to determine the implications of extending the NHS Breast Screening Programme (NHSBSP), to include routine invitations for women up to 69 years. All women aged 65–69 and registered with GPs in these areas received routine invitations for breast screening along with those aged 50–64. Overall uptake was 71% in women aged 65–69 compared with 78% in younger women, but was ≥ 90% in both groups who had previously attended within 5 years. Recall rates were lower for older women, but with a higher positive predictive value for cancer. The percentages of invasive cancer in different prognostic categories were similar in the 2 age groups. Older women took no longer to screen than younger women. The costs per woman invited or per woman screened were also similar to those for women aged 50–64, whilst the cost per cancer detected was some 34% lower in older women. Breast screening is as cost effective for women aged 65–69 as for those aged 50–64, with a higher cancer detection rate balancing shorter life expectancy. The proposed extension to the national programme will have considerable workforce implications for the NHSBSP and require additional resources. © 2001 Cancer Research Campaign

**Keywords**: breast cancer; screening; older women

The effectiveness of breast screening in women aged 50–64 has been well established by randomised trials, and recent analyses have shown that screening in women aged 65–69 is probably as effective in terms of reducing mortality from breast cancer as screening in women aged 50–64 (Chen et al, 1995).

The report of the Forrest committee (Forrest, 1986) in 1986, which led to the implementation of the NHS Breast Screening Programme in the United Kingdom, recommended that women aged 50–64 be invited for screening every 3 years, with women aged 65 and over being able to self-refer. The main reason for this distinction was concern over possible lower acceptance rates among older women, together with possible reduced cost-effectiveness due to decreased life expectancy in older women.

Population screening in a number of countries includes women up to age 69 or 70 (Shapiro et al, 1998). Some demonstration studies of inviting older women also showed that their uptake was only slightly lower than that for 50–64 year olds from the same locality, implying that reasonable uptakes could be achieved across the NHS as a whole (Hobbs et al, 1990; Hendry and Entwhistle, 1996; Horton-Taylor et al, 1996). The number of self-referrals in women aged 65 and over had increased to 65 032 by 1998/9, 44 811 of these being aged 65–69.

Demonstration studies at 3 sites were established by the Department of Health to determine the implications of extending the NHS Breast Screening Programme to women aged 65–69 by including this age-group in the routine invitation system. Findings from one of the sites after one year have previously been reported (Rubin et al, 1998). This paper presents the results of the evaluation of the full 3 years of all 3 sites. Decisions on the extension of the programme have been made on the basis of the results of these studies.

*Received 2 May 2001*
*Revised 17 July 2001*
*Accepted 17 July 2001*

### METHODS

The demonstration studies were scheduled to run for 3 years at each of 3 sites, with appointments for women aged 65–69 intermingled with those of women aged 50–64 and self-referrals of older women. As for women aged 50–64, those aged 65–69 were sent by post an invitation to attend at a specific date/time for mammographic screening. Those with an abnormality detected on the mammogram were sent an appointment for further assessment, which could include clinical examination, further mammographic views and ultrasound.

East Sussex, Brighton and Hove began screening for the study in May 1996, Nottingham in February 1997 and Leeds and Wakefield in April 1997. This timing means that women aged 65–67 had mostly been invited 3 years previously in the most recent screening round, but those aged 68–69 had mostly missed a round and would have been last invited 6 years previously.

The principal screening process and outcome measures were already routinely recorded at each demonstration site as part of the NHSBSP; these measures were analysed by age and time since last screen. Comparisons with equivalent data for the whole of England for the age-group 50–64 have been made to determine the representativeness of the demonstration sites.

In addition, pathology data were obtained from all 3 sites on the size, lymph node status and grade of screen-detected cancers in both younger and older women during the study period.

The Nottingham Prognostic Index (NPI) (Galea et al, 1992) was calculated from the available data using the formula: size (cm) × 0.2 + lymph node status (1–3) + grade. This index has been constructed and used to define 3 subsets of breast cancer patients up to age 70 with different prognosis. An index using a revised formula: size (cm) × 0.7 + grade for cases with unknown lymph node status was also calculated (Pinder S, Duffy S, personal communication).

The costs of an invitation, screen and assessment associated with breast screening in the UK setting estimated previously for women aged 50–64 for one-view mammography (Johnston et al, 1998) were used to form the basis of the unit cost estimates for women aged 65–69, uprated to 1999/2000 prices (NHS Executive, 1997) and to take account of the fact that a small proportion of women have more than one assessment visit (Johnston et al, 1996).

Data on the proportion of technical recall screens were available from all 3 sites. The cost of a technical recall was assumed to be the average of the cost of a screen taken on a mobile van and static unit.

To study possible variation in invitation costs with age, a survey was conducted to log telephone queries and appointment changes at each of the demonstration sites for all women aged 50–69 for one week each month over a period of a year, and the proportion in each age-group was calculated using a denominator estimated from the total number of women invited during the year.

If older women are less mobile and take longer to screen, this would affect throughput. Screening times and overall times spent in the unit were therefore measured for women aged 50–64 and 65–69 at each of the 3 demonstration sites, on a consecutive series of women until at least 50 women aged 65–69 had been screened. The timings took place at a mix of mobile and static sites during the winter months when the women were expected to be wearing more clothing.

The proportion of women assessed who had FNA cytology and/or core biopsy in the 2 age-groups was compared using data from standard returns. All other assessment procedures were assumed to be the same for both age-groups.

To estimate total costs, the numbers invited, screened and assessed in each age group for women previously screened were used, since in an extension to the programme the majority of women aged 65–69 would be in this category. The numbers invited, screened and assessed were summed across the 3 sites and multiplied by the respective unit cost. The effect of using figures for all women invited for screening (regardless of previous screening history), or of using those for women screened less than or equal to 5 years ago was addressed in the sensitivity analysis.

### RESULTS

Overall uptake in women aged 65–69 was 71%. Table 1 shows the uptake of screening by age (50–64 and 65–69) according to type of invitation. Results for England for women aged 50–64 for April 1997 to March 2000 are included for comparison. Uptake in previous non-attenders within the NHSBSP is generally low, and this was also observed in the demonstration studies. (A small percentage of women aged 65–69 were reported as receiving their first invitation; this may be due to women moving into a district whose previous screening history had not been added to the computer system.)

**Table 1** Uptake of screening (age groups 50–64 and 65–69)

| | | Total | | England 1997–2000 | |
|---|---|---|---|---|---|
| | | Invited | % uptake | Invited | % uptake |
| First invitation | | | | | |
| | 50–64 | 48 599 | 76 | 1 019 314 | 73 |
| | 65–69 | 1564 | 48 | | |
| Previous non-attenders | | | | | |
| | 50–64 | 25 874 | 24 | 519 168 | 23 |
| | 65–69 | 9628 | 19 | | |
| Previous screen ⩽5 years | | | | | |
| | 50–64 | 126 088 | 91 | 2 500 751 | 90 |
| | 65–69 | 23 846 | 90 | | |
| Previous screen > 5 years | | | | | |
| | 50–64 | 10 378 | 58 | 228 660 | 54 |
| | 65–69 | 14 937 | 77 | | |
| Total | 50–64 | 210 939 | 78 | 4 276 893 | 76 |
| | 65–69 | 49 975 | 71 | | |

For women previously screened within 5 years, uptake both in women 50–64 and 65–69 was high (91% and 90% respectively). For women with a screen more than 5 years previously, uptake was higher in women aged 65–69 (77%) than in those aged 50–64 (58%), reflecting the fact that many of the older women would not have been invited for 6 years, whereas the younger women would have been non-attenders at a previous invitation. Whilst there was variation in uptake levels between sites, with higher uptake in Nottingham and East Sussex, Brighton and Hove than in Leeds and Wakefield, the pattern was similar in all 3 sites.

Table 2 gives the outcomes of screening for the 3 sites combined for those women with a previous screen. Rates of referral for assessment were significantly lower in women aged 65–69 than in those aged 50–64 ($P < 0.0001$, $P = 0.04$ for women screened ≤5 years ago and >5 years ago, respectively). For each age category, referral rates were higher for women with a longer screening interval.

Invasive cancer detection rates were higher in women 65–69 than in those 50–64: 0.67% vs 0.41% for those previously screened within 5 years ($P < 0.0001$) and 0.71% versus 0.56% for those with a longer screening interval (NS, $P = 0.26$). The rates in women aged 65–69 were also higher than in those aged 60–64.

In-situ cancer detection rates were similar across age-groups for women screened within 5 years. For women with a longer screening interval comparison was difficult due to small numbers.

Uptake and outcome of screening of women aged 50–64 were similar to those for England as a whole, suggesting that the demonstration sites were representative of the national programme. There was no evidence of systematic differences between the results for the different years of the studies.

Benign diagnostic biopsy rates were similar across age-groups. The percentage of cancers with non-operative diagnosis was significantly higher ($P = 0.005$) in women aged 65–69 than in those aged 50–64 for those previously screened within 5 years.

The positive predictive value (PPV) of recall for assessment was consistently higher in the older age-group. For women previously screened within 5 years, the PPV for all cancers for women 65–69 was 29.0% compared to 15.6% for women 50–64. For women with a longer screening interval the percentages were 24.3% and 16.5%, respectively.

 *© 2001 Cancer Research Campaign*

**Table 2** Outcome of incident screens

***(i)* Women previously screened ≤ 5 years ago**

| | No of women screened | Referred for assessment | | In-situ cancers | | Invasive cancers | | Total cancers | | Benign biopsy | | Non-operative diagnosis | |
|---|---|---|---|---|---|---|---|---|---|---|---|---|---|
| | | No. | % | No. | % | No. | % | No. | % | No. | % | No. | % of cancers |
| 50–64 | 114 394 | 3913 | 3.4 | 139 | 0.12 | 472 | 0.41 | 611 | 0.53 | 62 | 0.05 | 488 | 79.9 |
| 60–64 | 42 655 | 1424 | 3.3 | 63 | 0.15 | 224 | 0.53 | 257 | 0.60 | 19 | 0.04 | 223 | 86.8 |
| 65–69 | 21 363 | 601 | 2.8 | 28 | 0.13 | 144 | 0.67 | 174 | 0.81 | 14 | 0.07 | 155 | 89.1 |
| England 1997–2000 | | | | | | | | | | | | | |
| 50–64 | 2 236 289 | 83 801 | 3.7 | 2105 | 0.09 | 9097 | 0.41 | 11 447 | 0.51 | 1731 | 0.08 | 9158 | 80.0 |
| 60–64 | 794 481 | 28 420 | 3.6 | 814 | 0.10 | 3930 | 0.49 | 4835 | 0.61 | 546 | 0.07 | 3953 | 81.8 |

***(ii)* Women previously screened > 5 years ago**

| | No of women screened | Referred for assessment | | In-situ cancers | | Invasive cancers | | Total cancers | | Benign biopsy | | Non-operative diagnosis | |
|---|---|---|---|---|---|---|---|---|---|---|---|---|---|
| | | No. | % | No. | % | No. | % | No. | % | No. | % | No. | % of cancers |
| 50–64 | 6070 | 255 | 4.2 | 8 | 0.13 | 34 | 0.56 | 42 | 0.69 | 12 | 0.20 | 31 | 73.8 |
| 60–64 | 3076 | 130 | 4.2 | 6 | 0.20 | 17 | 0.55 | 23 | 0.75 | 6 | 0.20 | 17 | 73.9 |
| 65–69 | 11 475 | 411 | 3.6 | 18 | 0.16 | 81 | 0.71 | 100 | 0.87 | 9 | 0.08 | 82 | 82.0 |
| England 1997–2000 | | | | | | | | | | | | | |
| 50–64 | 122 630 | 5968 | 4.9 | 183 | 0.15 | 667 | 0.54 | 870 | 0.71 | 150 | 0.12 | 688 | 79.1 |
| 60–64 | 55 707 | 2663 | 4.8 | 94 | 0.17 | 365 | 0.66 | 466 | 0.84 | 61 | 0.11 | 365 | 78.3 |

For a few cancers, the invasive status was not known so the total cancers will not always agree with the sum of in-situ and invasive cancers.

© 2001 Cancer Research Campaign

In all sites combined the percentage of invasive cancers with size <15 mm was 55% for women aged 65–69, compared with 63% for women 50–64, for women previously screened within 5 years. For women with a longer screening interval the percentages were 53% and 35% respectively (Table 3).

Table 4 gives the results for the Nottingham Prognostic Index (Galea et al, 1992) using the revised formula for cases with unknown lymph nodes status. The percentages falling into different prognostic categories in the 2 age-groups were similar.

Across all sites, the proportion of appointment queries or changes requested by women aged 65–69 was significantly less than that for those aged 50–64 (26.4% vs 29.7%, $P < 0.01$).

Table 5 shows the overall time the women spent at the screening unit and the time actually spent screening. The mean overall screening time and mean time attributable to the screen, at each of the demonstration sites, were not significantly different between the 2 age-groups ($P = 0.96$ overall times, $P = 0.10$ screen times).

The estimated unit cost of an invitation for both age-groups was £8.37; the cost of a screen was estimated to be £10.78 on a mobile van and £12.01 at a static unit. On the basis of information provided by the demonstration sites, it was assumed that East Sussex, Brighton and Hove screened all women on mobile vans, Leeds and Wakefield screened 88% of women on mobile vans and 12% at a static unit and that Nottingham screened 72% of women at a static site and 28% on mobile van.

The cost of a technical recall screen was assumed to be £11.40. Across the 3 sites the average proportion of technical recalls was 0.86% for women aged 50–64 years and 0.92% for women aged 65–69 years.

The estimated cost of an assessment was £53.09. The proportion of assessed women who had FNA cytology and/or core biopsy was greater for women aged 65–69 (40.0%) than for women aged 50–64 (31.4%) reflecting the increased PPV in older women. Whilst the cost of assessing a woman aged 65–69 may therefore be greater than that for a women aged 50–64, the cost per cancer detected will remain lower. In the absence of more accurate cost data, however, the cost of an assessment for both age-groups was assumed to be the same. The effect of doubling the cost of assessment for women aged 65–69 was addressed in the sensitivity analysis, but did not alter the main findings.

The total estimated cost to the breast screening programme of inviting 100 000 women aged 50–64 and 100 000 women aged 65–69 is shown in Table 6, and the cost per woman invited, per woman screened and per cancer detected in Table 7. There is little difference between the 2 age groups in terms of the cost per woman invited and the cost per woman screened. The total cost to the programme in terms of invitations, screens and assessments is slightly (3%) less per 100 000 women invited for women aged 65–69 years compared to women aged 50–64 years. The cost per cancer detected is 34% less for women aged 65–69 years. The overall findings remained similar for the sensitivity analyses.

**Table 3** Size of invasive cancers detected in incident screens (number of cases and % of women screened)

***(i)* Women screened ≤ 5 years ago**

| | < 10 mm | | ≥ 10–15 mm | | ≥ 15 mm | | Size not known | | Total | |
|---|---|---|---|---|---|---|---|---|---|---|
| | No. | % | No. | % | No. | % | No. | % | No. | % |
| 50–64 | 146 | 0.13 | 150 | 0.13 | 171 | 0.15 | 5 | 0.00 | 472 | 0.41 |
| 60–64 | 75 | 0.18 | 66 | 0.15 | 82 | 0.19 | 1 | 0.00 | 224 | 0.53 |
| 65–69 | 38 | 0.18 | 41 | 0.19 | 61 | 0.29 | 4 | 0.02 | 144 | 0.67 |
| England 1997–2000 | | | | | | | | | | |
| 50–64 | 2228 | 0.10 | 2781 | 0.12 | 3986 | 0.18 | 102 | 0.00 | 9097 | 0.41 |
| 60–64 | 997 | 0.13 | 1215 | 0.15 | 1676 | 0.21 | 42 | 0.01 | 3930 | 0.49 |

***(ii)* Women screened > 5 years ago**

| | < 10 mm | | ≥ 10–15 mm | | ≥ 15 mm | | Size not known | | Total | |
|---|---|---|---|---|---|---|---|---|---|---|
| | No. | % | No. | % | No. | % | No. | % | No. | % |
| 50–64 | 6 | 0.10 | 6 | 0.10 | 22 | 0.36 | 0 | 0.00 | 34 | 0.56 |
| 60–64 | 1 | 0.03 | 5 | 0.16 | 11 | 0.36 | 0 | 0.00 | 17 | 0.55 |
| 65–69 | 12 | 0.10 | 31 | 0.27 | 38 | 0.33 | 0 | 0.00 | 81 | 0.71 |
| England 1997–2000 | | | | | | | | | | |
| 50–64 | 147 | 0.12 | 189 | 0.15 | 319 | 0.26 | 12 | 0.01 | 667 | 0.54 |
| 60–64 | 80 | 0.14 | 107 | 0.19 | 170 | 0.31 | 8 | 0.01 | 365 | 0.66 |

**Table 4** Nottingham prognostic index (prevalent and incident screens) including cancers where the nodes were not sampled

| | Good | | Moderate | | Poor | | Not known | | |
|---|---|---|---|---|---|---|---|---|---|
| | No. | % | No. | % | No. | % | No. | % | Total |
| 50–64 | 411 | 58 | 218 | 31 | 53 | 7 | 25 | 4 | 707 |
| 65–69 | 155 | 56 | 90 | 33 | 18 | 7 | 13 | 5 | 276 |

**Table 5** Comparison of screen times by age group

| | Screen time (minutes:seconds) Mean (95% CI) (*n*) | Overall time (minutes:seconds) Mean (95% CI) (*n*) |
|---|---|---|
| 50–64 | 4:16 (4:08–4:23) ($n$ = 534) | 17:46 (16:55–18:38) ($n$ = 532) |
| 65–69 | 4:02 (3:48–4:16) ($n$ = 151) | 17:49 (16:23–19:15) ($n$ = 151) |

*© 2001 Cancer Research Campaign*

**Table 6** Total costs attributable to the screening programme per 100 000 women invited

| Age group | Cost of invitations (£) | Cost of screens[a] (£) | Cost of assessments (£) | Total (£) |
|---|---|---|---|---|
| 50–64 | 837 000 | 974 497 | 162 150 | 1 973 647 |
| 65–69 | 837 000 | 933 669 | 138 533 | 1 909 202 |

[a]Includes cost of technical recall screens.

**Table 7** Total cost per woman invited[a], screened and cancer detected

| Age group | Total cost per woman invited (£) | Total cost per woman screened (£) | Total cost per cancer detected (£) |
|---|---|---|---|
| 50–64 | 19.74 | 22.36 | 4124.59 |
| 65–69 | 19.09 | 22.54 | 2702.36 |

[a]Costs associated with invitations, screens and assessments.

### DISCUSSION

One of the original concerns about inviting older women for screening was their possible lower uptake. In these demonstration studies uptake in women aged 65–69 being invited for re-screening was high, and similar to that for younger women. Reasons for this may include the fact that the women included should all have been invited at least once previously within the NHSBSP and would therefore be accustomed to the idea of routine screening. This may contrast with earlier studies where many of the women were receiving their first invitation at an older age. Hobbs et al (1990) found an uptake of 62.2% in women aged 65–69 compared with 69.3% in those aged 50–64 at prevalent screen, whilst Horton Taylor et al reported an uptake of 34.5% in women 65–69 compared with 38.9% in younger women in an inner city area with low uptake (Horton-Taylor et al, 1996).

Future national uptake rates might be expected to be even higher if invitations for older women were to become national policy because of the accompanying publicity. In the Netherlands, where women are routinely invited up to age 74, the attendance in previous attenders was 90.5% in women aged 65–69 and 78.1% in those aged 70–74, compared with 92.5% in women aged 60–64 (National Evaluation Team for Breast Cancer Screening, 2000).

For comparison between age-groups and extrapolation to the screening programme as a whole the results of incident screens are most appropriate. Observed invasive cancer detection rates were higher for older women in line with those expected given the increasing incidence with age. In contrast, detection rates of in-situ cancers were no higher in the older age-groups. Referral rates for assessment were lower in older women; this is in contrast to the Netherlands, which has generally low referral rates, where the rate at incident screens in women aged 65–69 was 0.46% compared with 0.31% in those aged 50–64 (Fracheboud et al, 1998), although the PPV of referral was still higher in the older age-group.

Thus in terms of uptake and cancer detection, invitation to screening would appear to have performed at least equally well here in women 65–69 as in younger women. Characteristics of screen-detected tumours in the 2 age-groups support the prediction that a similar reduction in breast cancer mortality would be achieved by screening older women to that resulting from screening women 50–64. Similar findings have been reported from the Netherlands screening programme (van Dijck et al, 1996).

Estimates of the cost of screening women aged 65–69 compared to those aged 50–64 show that the cost per cancer detected is approximately 34% lower. Our analysis assumes that the unit cost of an invitation, screen and assessment for a women aged 65–69 was the same as that for a woman aged 50–64. There was no evidence to suggest that the unit cost of an invitation or screen would be any more for a woman aged 65–69 compared to a woman aged 50–64, and indeed the cost of an invitation may well be less. Doubling the cost of assessment for women aged 65–69 did not alter the main findings.

Biopsy and treatment costs were not quantified. However, the results suggested a similar proportion of women invited for screening would be referred for biopsy in both age-groups. A greater number of cancers were detected in women aged 65–69 compared to women aged 50–64, implying the total cost associated with treatment will be higher per 100 000 women invited for screening for women aged 65–69. Most of these women would, however, otherwise require treatment for symptomatic disease within a few years.

A crude comparison of the average cost per life-year gained can be made by assuming the same proportion of deaths from breast cancer is prevented, in women with screen-detected invasive cancer, regardless of the age-group. The ratio of deaths prevented in the 50–64 age group to the 65–69 age group is 4:7 (ie. the same as that of the cancer detection rates). If, for example, the average life years gained in the 2 age groups are 15 and 8.5 respectively, the ratio of life years gained is $(4/7) \times (15/8.5) = 1.01$. The increased cancer detection rate in older women then approximately balances the reduction in life-years gained, so that cost per life-year gained in each age-group would be similar.

These studies were conducted in women aged 65–69; an extension of the NHSBSP would more logically cover those aged 65–70, so that all women receive 2 additional invitations, and a proposal for such an extension has recently been announced in the NHS Cancer Plan with all programmes starting to invite older women by 2004 (Department of Health, 2000). This is a challenging expansion that will necessitate additional buildings, equipment and staff. For England as a whole there are 1.35 million women aged 65–70; extending the invitation system as proposed

© 2001 Cancer Research Campaign 

will result in approximately 450 000 invited each year with 320 000 or more of these attending each year, compared with the 45 000 women aged 65–69 currently self-referring. Extrapolating from the experience of one of the demonstration sites, this would require around £13.5 million per annum, including staff costs of an additional 25 whole time equivalent radiologists and 187 radiographers and non-staff costs of £5.9 million per annum. This figure can only be indicative as it is based on one centre and non-staff costs in particular are known to vary between centres (Johnston et al, 1996). This is higher than the figure of £8.6 million obtained using the estimated cost per woman invited derived earlier where an opportunity cost approach was used to estimate the unit costs, whereby the resources required for each screening activity were quantified and valued (Johnston et al, 1996). The latter will not have included aspects of the programme such as quality assurance and national co-ordination. Recommended staffing levels have also increased since the original estimates were derived. The findings relating to the cost differences for women aged 65–69 compared to women aged 50–64 still hold even if the unit cost of a screen were doubled such that the cost per woman invited is £27.86 for women aged 65–69 and £29.87 for women aged 50–64 and the total resources required per annum to screen women aged 65–69 is £12.4 million.

In conclusion, including women up to age 70 in the invitation system is likely to be as cost-effective as for women aged 50–64, and no major logistical difficulties with screening older women were identified. The proposed inclusion of older women in the invitation system by the NHSBSP over the next 5 years has considerable workforce implications, which new skill mix arrangements about to be piloted will help to address.

### ACKNOWLEDGEMENTS

This study was funded by the Department of Health. The views expressed in this publication are those of the authors and not necessarily those of the Department of Health.

We would like to thank the staff involved in East Sussex, Brighton and Hove, Leeds and Wakefield and Nottingham for their invaluable assistance in producing this report, both in obtaining and supplying the necessary data and for commenting on numerous drafts. We would also like to thank Kathy Johnston for her help with the economic evaluation. Much of this work was conducted whilst Jackie Brown was employed as a Senior Research Fellow in the Health Economics Research Group at Brunel University.

### REFERENCES

Chen HH, Tabar L, Fagerberg G and Duffy SW (1995) Effect of breast cancer screening after age 65. *J Med Screening* **2**: 10–14

Department of Health. *The NHS Cancer Plan. A plan for investment. A plan for reform*. 1–98. 2000. London

Forrest APM (1986) *Breast cancer screening: report to the health ministers of England, Wales, Scotland and Northern Ireland*. London: HMSO

Fracheboud J, de Koning HJ, Beemsterboer PM, Boer R, Hendriks JH, Verbeek AL, van Ineveld BM, de Bruyn AE and van der Maas P (1998) Nation-wide breast cancer screening in the Netherlands: results of initial and subsequent screening 1990–1995. *Int J Cancer* **75**: 694–698

Galea MH, Blamey RW, Elston CE and Ellis IO (1992) The Nottingham Prognostic Index in primary breast cancer. *Breast Cancer Res Treat* **22**: 207–219

Hendry PJ and Entwhistle C (1996) Effect of issuing an invitation for breast cancer screening to women aged 65 to 69. *J Med Screening* **3**: 88–89

Hobbs P, Kay C, Friedman EH, St. Leger AS, Lambert C, Boggis CR, Howard TM, Owen AW and Asbury DL (1990) Response by women aged 65–79 to invitation for screening for breast cancer by mammography: a pilot study. *BJM* **301**: 1314–1316

Horton-Taylor D, McPherson K, Parbhoo S and Perry N (1996) Response of women aged 65–74 to invitation for screening for breast cancer by mammography: a pilot study in London, U.K. *J Epidemiol Community Health* **50**: 77–80

Johnston K, Gerard K, Morton A and Brown J (1996) NHS costs for the Breast Screening Frequency and Age Trials. *HERG Discussion Paper November* No**. 16**:

Johnston K, Gerard K and Brown J (1998) Analyzing center selection bias in a breast screening trial. *Int J Technol Assess Health Care* **14**: 494–504

National Evaluation Team for Breast Cancer Screening. Llandelijke evaluatie van bevolkingsonderzoek naar borstkanker in Nederland. 2000. Rotterdam, Erasmus University, Catholic University, Nijmegen.

NHS Executive (1997) *Health Service Cost Index (HCHS Specific Price Inflation)*. Leeds: Department of Health

Rubin G, Garvican L and Moss S (1998) Routine invitation of women aged 65–69 for breast cancer screening: results of the first year at the East Sussex Brighton & Hove Screening Service pilot site. *BMJ* **317**: 388–389

Shapiro S, Coleman EA, Broeders M, Codd M, de Koning H, Fracheboud J, Moss S, Paci E, Stachenko S and Ballard-Barbash R (1998) For the International Breast Cancer Screening Network (IBSN) and the European Network of Pilot Projects for Breast Cancer Screening. Breast cancer screening programmes in 22 countries: current policies, administration and guidelines. *Int J Epidemiol* **27**: 735–742

van Dijck JAAM, Verbeek AL, Hendricks JHCL, Holland R and Mravunac M (1996) Mammographic screening after the age of 65 years: early outcomes in the Nijmegen programme. *Br J Cancer* **74**: 1838–1842

*© 2001 Cancer Research Campaign*
